# Effect of the Modification of the Number of Players, the Size of the Goal, and the Size of the Field in Competition on the Play Actions in U-12 Male Football

**DOI:** 10.3390/ijerph17020518

**Published:** 2020-01-14

**Authors:** Antonio García-Angulo, José Manuel Palao, José María Giménez-Egido, Francisco Javier García-Angulo, Enrique Ortega-Toro

**Affiliations:** 1 Department of Physical Activity and Sport, Faculty of Sport Science, University of Murcia, Regional Campus of International Excellence “Campus Mare Nostrum”, 30720 Murcia, Spain; josemaria.gimenez@um.es (J.M.G.-E.); franciscojavier.garcia19@um.es (F.J.G.-A.); 2 Murcia Football Federation (FFRM), 30008 Murcia, Spain; 3 Health, Exercise science and Sport Management Department, University of Wisconsin (Parkside), Kenosha, WI 53144, USA; palaojm@gmail.com

**Keywords:** children, player development, sport, competition, rules, technique, tactics

## Abstract

A player’s sports development involves a long process. The modification of rules for youth players seeks to adapt the sport to the child and his/her development. The manipulation of rules affects the technical and tactical skills demonstrated by players and, therefore, their development. The objective of this study was to analyse the effect of a reduction in the number of players (from 8 per team to 5 per team), the size of the goal (from 6 × 2 m to 3 × 2 m) and the playing space (from 58 × 38 m to 38 × 20 m), on the technical and tactical actions in youth football players. A quasi-experimental A-B-A design was implemented to assess the effect of the rule changes. The players (*n* = 40) played three tournaments using two competition formats (official rules, modified rules, and official rules). The results show that the use of the modified rules generated a greater number and variability in the technical–tactical actions, a greater number of actions with teammates in the pass line, a greater continuity in the game, a greater number of attack and defence actions in areas close to the goal, and favours team play. The experimental format fits the players’ individual progression better (U-12) as well as the players’ and teams’ collective development, and it will allow players to evolve from the individual development of previous stages.

## 1. Introduction

Football is a late specialization sport with a long development process [1,2]. During this process, players go through different phases in relation to their biological age and training. For players, the goal of the process is to have appropriate experiences with regard to their level of maturity and skill. The progressive accumulation of these experiences through competition and training is what allows players to increase their performance [3]. For this reason, each stage of the process needs specific game rules that allow for the long-term athletic development (LTAD) of the players [4]. The modification of the rules seeks to adapt the sport to the child and his/her development. The rule modifications seek to increase children’s participation through actions that are appropriate for their physical, technical, and psychological characteristics [5,6,7]. The modification of structural elements of football, such as scoring systems, affects players’ mental fatigue [8]. In each country or region, federations or institutions establish competition formats and rules adapted to the players within the formation. For example, in Spain, the national federation recommends competition formats for each age group, and the territorial federations establish the specific format for each category. However, there is no consensus on the most appropriate format for each age group. There are different proposals focused on the modification of the number of players, the dimensions of the pitch and goals, the playing time, and the manipulation of the offside rule [9,10,11]. However, the incidence of these modifications on the actions of the game and the adequacy of the game format in the development of the youth player is unknown.

In the specialized bibliography, it is possible to find different theoretical models for the development of the youth football player [12]. These models evolve in the number of players, and the size of the field and goal, progressively (e.g., 3 vs. 3 (U-8), 5 vs. 5 (U-10) and 9 vs. 9 (U-13)). These formats seek to reduce the impact of age-group changes and achieve greater player participation by adapting the rules [12,13,14]. There are few experimental studies that analyse the effect of rule changes on the player’s LTAD. Most of the available information comes from descriptive observational studies. Regarding the manipulation of the number of players, the observational studies show that a reduction in the number of players compared to the 11 vs. 11 format resulted in a greater number of technical–tactical offensive actions [9,10,13,14], greater effectiveness in offensive actions [8], and more depth and breadth in offensive game actions [9,13,14]. In relation to the manipulation of the dimensions of the field, studies have found the importance of the dimensions of the field on players’ technical–tactical behaviours [15]. An increase in the area per player involves a greater number of technical–tactical offensive actions and greater efficiency in the offensive actions and the development of the game [9,14]. The variables of interaction and proximity between the players, associated with the dimensions of the field, influence the number of shots and passes [16]. These modifications also affect specific actions done by the players with the ball, such as dribbling [17]. As far as the size of the goal is concerned, a bigger goal involves a higher number of shots on goal [10], and a smaller goal involves a higher number of interventions by the goalkeeper [18].

The use of a competition format not adapted to the characteristics of players could have a negative impact on their training process and their LTAD [19,20]. The low number of experimental studies makes it difficult to assess the real effect of the regulatory changes and which is the most suitable competition format for each age-group for LTAD in football. The information currently available shows that a reduction in the number of players, the pitch, and the size of the goals compared to the normative competition could result in an improvement in the technical–tactical parameters at a qualitative and quantitative level [9,10,13,14]. Given that most common regulatory competitions in Spain are of the football-8 and football-7 formats, with a playing area of 171.42 m^2^ and 150 m^2^, respectively, per player, and with goals measuring 6 × 2 m, a smaller format could contribute to the development of competitions that allow for a better development of youth football players according to their maturity and sport skills. Knowing the incidence of these rule changes will allow different stakeholders to adapt the game to the youth football players. The objective of this study was to analyse the effect of a reduction in the number of players (from 8 vs. 8 to 5 vs. 5), the size of the goal (from 6 × 2 m to 3 × 2 m) and the playing space (from 58 × 38 m to 38 × 20 m), on the technical and tactical actions in youth football players.

## 2. Materials and Methods

### 2.1. Design

A quasi-experimental A-B-A design was implemented to assess the effect of the rule changes. The design conditions moved from official rules (situation A, no changes in the rules), change in the official rules (situation B, modified rules), and official rules (situation A). The A-B-A type design is characterized by two control phases that give the study a higher degree of internal validity than the classic A-B type designs [21]. The players studied played the tournaments with their own teams.

### 2.2. Participants

The sample consisted of 40 male under-12 football players belonging to four amateur male teams (10 players per team) of the U-12 age group. The characteristics of the players were the following: average age (11.73 ± 0.43 years); training sessions per week (2.50 ± 0.57 sessions); session time (1.37 ± 0.44 h); hours of training per week (3.27 ± 0.65 h), and years of experience (2.93 ± 1.15 years). The guardians of the players were informed of the study and provided written consent. The players played three tournaments using two competition formats (official rules, modified rules, and official rules) and using a pre-established system of substitutions. A total of 8697 ball possession actions taken by the players in 24 matches were analysed. The study was approved by the University Ethics Committee of the principal authors with ID 1944/2018.

### 2.3. Variables

The independent variable was the game format. There were two levels: official rules and modified rules. The differences between the official and modified rules were the following: size of the field (58 × 38 m vs. 38 × 20 m), number of players per team (8 per team vs. 5 per team), and goal size (6 × 2 m vs. 3 × 2 m). Table 1 shows the rules that were used in both competition formats. The first and third tournaments were played according to the official state rules for U-12 competitions established by the football federation (football-8). In the second tournament, the modification of the official U-12 football rules (football-5) was applied.

The dependent variables were: the technical–tactical actions by which the player obtained the ball (steal, clearance from a player, rebound, pass interception, set-piece kick, teammate pass, pass after throw-in, pass after set piece, and throw-in); actions done when the team has ball possession (basic collective tactical actions, dribbles, number of contacts when driving, and type of dribble); and actions which end teams’ ball possession (throw-in pass, shot off target, shot stopped by the goalkeeper, ball lost, clearance from a player, out of bounds, and other actions); the ball height in which the possession starts and ends; the body surface used to start and end the possession; distance from nearest opponent; teammates supporting; pressure lines surpassed with ball; and field area. The variables registered are part of the observation instrument (observation instrument for technical and tactical actions of the offence phase in soccer) that was designed and validated by Ortega-Toro, García-Angulo, Giménez-Egido, García-Angulo, and Palao [22].

### 2.4. Procedure

The data were recorded in three tournaments that were played in a period of three weeks (one week between tournaments). The tournaments were played after the end of the official regular season and on weekends. All tournaments were played at the same time of day and in similar weather conditions. In total, 24 matches were played in the three tournaments (six matches in the first tournament; 12 matches in the second tournament; and six matches in the third tournament). The competition system was round robin. The order of the confrontations was the same in the different tournaments. Before the tournaments, a pre-established system of substitutions was determined, in order to establish an equitable distribution of minutes per player (1st tournament: 25.45 ± 1.45; 2nd tournament: 32.00 ± 2.48; and 3rd tournament: 25.45 ± 1.45). In the second tournament (situation B, modified rules), each team played the same number of matches as in the first and third tournaments, but each team was divided into two sub-teams. This allowed for the playing of two simultaneous matches on adjacent soccer fields, with two 20-min periods, played on a 38 × 20 m soccer field, with five players plus one outfield player for substitutions, and two goals on each 38 × 20 m field. After the first game between sub-teams, the other sub-teams’ match was played. The result of the matches was calculated from the overall goals scored in both matches. In all the tournaments, a 10-min half-time period was established between each period of the same match. Once a match was finished, five minutes were established to start the next match.

The actions developed by the players were recorded with two fixed digital cameras from an elevated rear view. The actions were recorded and analysed by two trained observers (with a Masters degree in Sports Science and at least 5 years of experience in match analysis and soccer). The observers were trained with the observation instrument before beginning the study. After the training period, the inter- and intra-observer reliability were calculated. To calculate the intra-observer reliability, another researcher was used as a reference. The researcher held a sports science degree and had more than ten years of experience in sports analytics. The reliability of the observers was measured before and after the observation. The lowest level of interobserver reliability was 0.83, and the lowest level of intra-observer reliability was 0.92 (Kappa index).

### 2.5. Data Analysis

Descriptive (means and standard deviation) and inferential statistics of the data were calculated. To measure the difference between different tournaments, an analysis of variance for repeated measures was calculated. Mauchly’s test of sphericity and Pillai’s trace were used. Bonferroni post hoc analysis was used. The level of significance was set at *p* < 0.05. To measure the magnitude of the effect size, the eta square (*η^2^*) was used, using the following classification [23]: no effect (*η^2^* < 0.04), minimum effect (0.04 < *η^2^* < 0.25), moderate effect (0.25 < *η^2^* < 0.64) and strong effect (*η^2^* > 0.64). The statistical analysis was completed with SPSS software (version 24.0, IBM, Chicago, IL, USA).

## 3. Results

Regarding the way players obtained the ball (Table 2), the results show statistically significant differences between tournaments in obtaining the ball after a clearance from a player (F_2,6_ = 8.610, *p* = 0.001, *η^2^* = 0.330). Statistically significant differences were observed between Tournament 1 and Tournament 2 (*p* = 0.001). There were no significant differences between Tournaments 1 and 3 (*p* = 0.229) or between Tournaments 2 and 3 (*p* = 0.100). The effect size on this variable was moderate. Significant differences were found when the ball was obtained by intercepting a pass (F_2,6_ = 7.330, *p* = 0.002, *η^2^* = 0.295), between Tournament 1 and Tournament 2 (*p* = 0.001). These differences had a moderate effect size. Statistically significant differences were found in the number of balls obtained by a pass from a teammate. There was a significantly higher number of passes in Tournament 2 (F_2,6_ = 8.961, *p* = 0.001, *η^2^* = 0.339). A post hoc analysis shows differences between Tournament 1 and Tournament 2 (*p* < 0.001). Tendencies toward significance were found between Tournament 2 and Tournament 3 (*p* = 0.061), and no differences were found between Tournament 1 and Tournament 3 (*p* = 0.486). These differences had a moderate effect size. Significant differences were found regarding throw-ins (F_2,35_ = 16.428, *p* < 0.001, *η^2^* = 0.484) and after a throw-in (F_2,6_ = 9.043, *p* = 0.001, *η^2^* = 0.341). Tournament 2 had significantly higher values than Tournament 1 for both variables (*p* < 0.001). These differences had a moderate effect size for both variables.

Related to the way that the ball was obtained (Table 3), statistically significant differences were found for all the heights at which the ball is obtained: ground level (F_2,35_ = 6.260, *p* = 0.005, *η^2^* = 0.263), middle-height ball (F_2,35_ = 6.986, *p* = 0.003, *η^2^* = 0.285), and a high ball (F_2,35_ = 13.900, *p* < 0.001, *η^2^* = 0.443). Tournament 2 had significantly higher occurrence of high balls than Tournament 1 and Tournament 3. These differences had a moderate effect size. Statistically significant differences were found in obtaining the ball with the foot (F_2,35_ = 10.888, *p* < 0.001, *η^2^* = 0.384), with the hand (F_2,35_ = 7.079, *p* = 0.003, *η^2^* = 0.288), and with the head (F_2,35_ = 13.382, *p* < 0.001, *η^2^* = 0.433). Tournament 2 had a higher occurrence than Tournament 1 (*p* < 0.003) and Tournament 3 (*p* = 0.010). These differences had a moderate effect size. Statistical differences were found in the variable very close distance from the nearest opponent (F_2,35_ = 7.052, *p* = 0.003, *η^2^* = 0.287), in close distance (F_2,35_ = 5.844, *p* = 0.006, *η^2^* = 0.250), in near distance (F_2,35_ = 6.843, *p* = 0.003, *η^2^* = 0.281), and in distant distance (F_2,35_ = 36.315, *p* < 0.001, *η^2^* = 0.675). Tournament 2 had a higher occurrence of near and far away distances than Tournament 1 and Tournament 3. The effect size of this variable was moderate for the very close, close, and near distances and was strong for the far away distance. Statistical differences were found in the situation where a teammate was supporting the player with the ball between the tournaments (F_2,35_ = 13.985, *p* < 0.001, *η^2^* = 0.444). Tournament 2 had a higher occurrence than Tournament 1 (*p* < 0.001) and Tournaments 3 (*p* = 0.044). The effect size of this variable was moderate.

**Figure 1:**
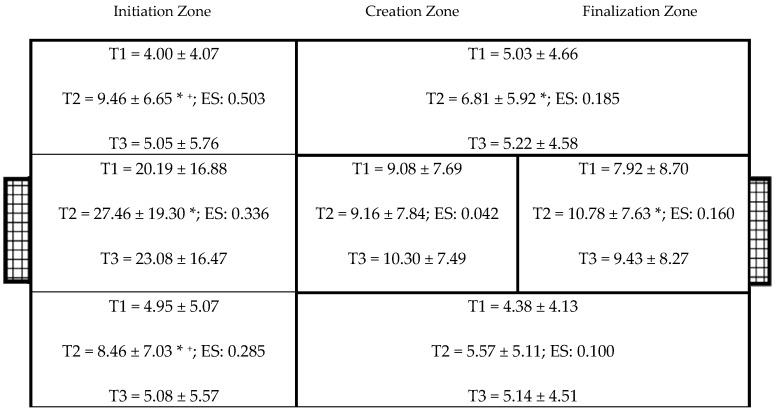
Zones where the player receives the ball. Legend: * Statistically significant differences between Tournament 1 (T1) and Tournament 3 (T3); ^+^ Statistically significant differences between Tournament 2 (T2) and Tournament 3 (T3); ES: effect size (*η^2^*).

Figure 1 shows the zones in which the player obtained possession of the ball. Statistically significant differences were found in the left initiation zones (F_2,35_ = 17.691, *p* < 0.001, *η^2^* = 0.503). Tournament 2 had a higher occurrence for this than Tournament 1 (*p* < 0.001) and Tournament 3 (*p* < 0.001). The effect size of this variable was moderate. In the central zone (F_2,35_ = 8.875, *p* = 0.001, *η^2^* = 0.336), Tournament 2 had a higher occurrence than Tournament 1 (*p* = 0.001). The effect size of this variable was moderate. In the right initiation zone (F_2,35_ = 6.967, *p* = 0.003, *η^2^* = 0.285), Tournament 2 had a higher occurrence than Tournament 1 (*p* = 0.002) or Tournament 3 (*p* = 0.011). The effect size of this variable was moderate. Statistically significant differences were found in the left creation–finalization zone (F_2,35_ = 3.968, *p* = 0.028, *η^2^* = 0.185). Tournament 1 had a higher occurrence for this than Tournament 2 (*p* = 0.025). The effect size of this variable was minimal. Statistically significant differences were found in the central finishing zone (F_2,35_ = 3.337, *p* = 0.047, *η^2^* = 0.160). Tournament 1 had a higher occurrence for this than Tournament 2 (*p* = 0.045). These differences had a moderate effect size.

Regarding the actions performed by the players with the ball (Table 4), statistically significant differences in the use of basic collective tactical actions were found (F_2,6_ = 12.022, *p* < 0.001, *η^2^* = 0.414.). Tournament 2 had a significantly higher occurrence of the non-use of collective tactical actions than Tournaments 1 and 3 (*p* < 0.001). Tournament 2 had a significantly higher occurrence of the use of the wall than Tournament 3 (*p* = 0.002). In both variables, these differences had a moderate effect size. Significant differences were found in other collective tactical actions (F_2,34_ = 11.770, *p* < 0.001, *η^2^* = 0.409). Tournament 2 had a higher use of penetration actions than Tournament 1 (*p* < 0.001) and Tournament 3 (*p* < 0.001). The effect size was moderate. The use of no dribbling was statistically significant and higher in Tournament 2 than in Tournaments 1 and 3 (F_2,6_ = 16.352, *p* < 0.001, *η^2^* = 0.483). These differences had a moderate effect size. In the analysis of the number of contacts whilst dribbling the ball, the results show differences in the number of actions that occur without dribbling (F_2,35_ = 12.069, *p* < 0.001, *η^2^* = 0.408) and while dribbling the ball with two contacts (F_2,35_ = 6.547, *p* = 0.004, *η^2^* = 0.272). Tournament 2 had a significantly higher occurrence than Tournament 1 (*p* = 0.009) and 3 (*p* = 0.009, no dribbling). In both variables, the effect size was moderate. The results with regard to the type of dribbling show differences in actions, in which players did not dribble the ball (F_2,35_ = 11.973, *p* < 0.001, *η^2^* = 0.406). Tournament 2 had a higher occurrence than Tournaments 1 (*p* < 0.001) and 3 (*p* = 0.040). Tournament 3 had a higher occurrence than Tournament 1 (*p* = 0.023). The size effect was moderate. Tournament 2 had a significantly higher use of the timing actions than Tournament 1 (F_2,35_ = 7.858, *p* = 0.002, *η^2^* = 0.310). Significant differences were found in the use of penetration actions (F_2,35_ = 25.550, *p* < 0.001, *η^2^* = 0.593). Tournament 2 had a significantly higher use of penetration actions than Tournament 1 (*p* < 0.001) and Tournament 3 (*p* < 0.001). Tournament 3 had a significantly higher use of penetration actions than Tournament 1 (*p* = 0.028). In both, the effect size was moderate. The actions in which no pass lines were surpassed were significantly higher in Tournament 2 than in Tournament 1 (F_2,35_ = 43.545, *p* < 0.001, *η^2^* = 0.713). The effect size was strong. The actions in which a player passed one defensive line with a pass were significantly higher in Tournament 2 than in Tournament 1 (F_2,35_ = 5.243 ^a^, *p* = 0.010, *η^2^* = 0.231). The effect size was moderate.

Regarding the way the ball possession ends (Table 5), significant differences between tournaments were found in the actions of losing the possession with a pass to a teammate (F_2,35_ = 12.578, *p* < 0.001, *η^2^* = 0.418). Tournament 2 had a significantly higher occurrence than Tournament 1 (*p* = 0.001). Tournament 3 had a significantly higher occurrence than Tournament 1 (*p* = 0.002). The effect size of this variable was moderate. Significant differences between tournaments were found in the number of shots stopped by the goalkeeper (F_2,35_ = 16.552, *p* < 0.001, *η^2^* = 0.486). Tournament 2 had a significantly higher occurrence than Tournament 1 (*p* <.001) and Tournament 3 (*p* = 0.001). The effect size of this variable was moderate. There were differences between tournaments in the number of balls lost (F_2,35_ = 4.017, *p* = 0.027, *η^2^* = 0.187). Tournament 2 had a significantly higher occurrence than Tournament 1 (*p* = 0.027) and Tournament 3 (*p* = 0.026). The effect size of this variable was minimal. Significant differences between tournaments were found in the number of clearances (F_2,35_ = 17.687, *p* < 0.001, *η^2^* = 0.503). Tournament 2 had a significantly higher occurrence than Tournament 1 (*p* < 0.001) and Tournament 3 (*p* = 0.013). The effect size of this variable was moderate. Significant differences between tournaments were found in out of bounds (F_2,35_ = 7.755 ^a^, *p* = 0.002, *η^2^* = 0.307). Tournament 2 had a significantly higher occurrence than Tournament 1 (*p* < 0.004) and Tournament 3 (*p* = 0.001). The effect size was moderate. Significant differences between tournaments were found in other actions (F_2,35_ = 6.728 ^a^, *p* = 0.003, *η^2^* = 0.278). Tournament 2 had a significantly higher occurrence than Tournament 1 (*p* < 0.002). The effect size was moderate.

**Figure 2:**
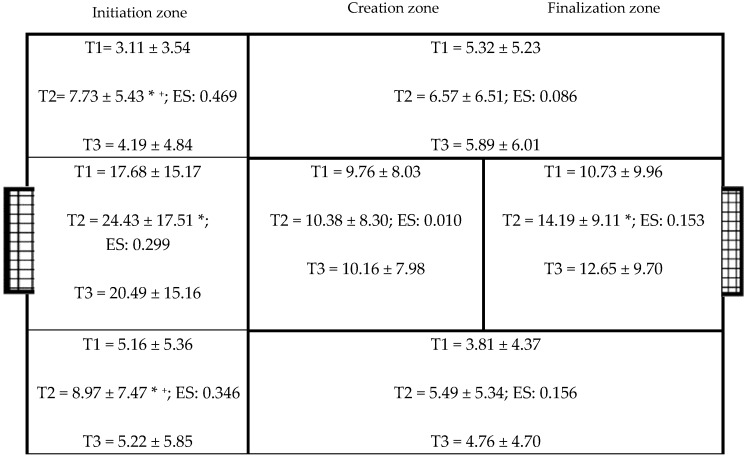
Zone where possession ends * Statistically significant differences between T1 and T3. ^+^ Statistically significant differences between T2 and T3. ES: effect size (*η^2^*).

Related to the zones in which the possession of the ball ends (Figure 2), statistically significant differences were found in the left initiation zone (F_2,35_ = 15.449, *p* < 0.001, *η^2^* = 0.469). Tournament 2 had a significantly higher occurrence than Tournament 1 and Tournament 3 (*p* < 0.001). Statistically significant differences were found in the central initiation zone (F_2,35_ = 7.450, *p* = 0.002, *η^2^* = 0.299). Tournament 2 had a significantly higher occurrence than Tournament 1 (*p* = 0.002). Statistically significant differences were found in the right initiation zone (F_2,35_ = 9.279, *p* = 0.001, *η^2^* = 0.346). Tournament 2 had a significantly higher occurrence than Tournament 1 (*p* < 0.001) and Tournament 3 (*p* < 0.002). The effect size of these three variables was moderate. A trend towards significance was found in the central end zone (F_2,35_ = 3.164, *p* = 0.055, *η^2^* = 0.153). Tournament 2 had a more significant tendency to have a higher occurrence than Tournament 1 (*p* = 0.059). The effect size of these three variables was minimal.

Regarding the characteristics of the technical–tactical actions in which the player lost possession of the ball (Table 6), statistically significant differences were found in the middle ball height (F_2,35_ = 35.226, *p* < 0.001, *η^2^* = 0.668), and in high balls (F_2,35_ = 11.602, *p* < 0.001, *η^2^* = 0.399). Tournament 2 had a higher occurrence than Tournament 1 and Tournament 3 (*p* < 0.001). The effect sizes of these three variables were strong and moderate, respectively. A significantly higher number of actions ended with the foot (F_2,35_ = 9.459, *p* = 0.001, *η^2^* = 0.351), with the hands (F_2,35_ = 13.470, *p* < 0.001, *η^2^* = 0.435), and with the head (F_2,35_ = 14,622, *p* < 0.001, *η^2^* = 0.455). Tournament 2 had a significantly higher occurrence than Tournament 1 (*p* < 0.001). In the actions that ended with the head, Tournament 2 had a significant higher occurrence than Tournament 3 (*p* < 0.001). The effect size of these three variables was moderate. Statistically significant differences were found in the actions that ended at a very close distance to the opponent (F_2,35_ = 6.503, *p* = 0.004, *η^2^* = 0.27), and at close distance (F_2,35_ = 13.409, *p* < 0.001, *η^2^* = 0.434). Tournament 2 had a significantly higher occurrence than Tournament 1 (*p* < 0.001). In the actions that ended at a very close distance to the opponent, Tournament 2 had a significantly higher occurrence than Tournament 1 (*p* < 0.003) and Tournament 3 (*p* < 0.042). The effect size was moderate in both variables. Statistically significant differences were found in the presence of a supporting partner between tournaments (F_2,35_ = 10.383, *p* < 0.001, *η^2^* = 0.372). Tournament 2 had a significantly higher occurrence than Tournament 1 (*p* < 0.001) and 3 (*p* = 0.042). The effect size was moderate. Statistically significant differences were found in situations in which a pressure line was not surpassed (F_2,35_ = 10.938, *p* < 0.001, *η^2^* = 0.385) and in situations in which the pressure line was surpassed (F_2,35_ = 13.078, *p* < 0.001, *η^2^* = 0.428). Tournament 2 had a significantly higher occurrence than Tournament 1 (*p* < 0.001) and 3 (*p* < 0.004). The effect size was moderate.

## 4. Discussion

The objective of this study was to analyse the effect of a reduction in the number of players, the size of the goal, and the playing space on the technical and tactical actions in youth male football players. An experimental tournament was carried out to test the implication of these rule changes on U-12 players. The changes in field and goal size and number of players resulted in an increase in the individual and collective actions done by the players. The experimental rules involved greater variability in the type of actions done by players related to the contact height, the contact surface, and the distance of the opponent. The rule changes led to a higher number of passes and actions when a teammate supported the player with the ball. These increments could be due to the increase in the players per square meter, which reduced the possibilities to progress by dribbling the ball, which were higher under the official rules. The experimental rules involved a higher use of the lateral zones of the field, and a higher number of shots, passes, and interceptions. This combination, more passes, and the use of more areas of the field created more space on defence, a higher number of offensive actions, and higher collective participation in the experimental format. From the perspective of the players’ experience, the proposed rules involved higher participation and higher variability. This could help to improve tactical thinking and the ability to try different solutions for game problems, etc. [24]. The results confirm the findings of previous observational studies that showed a higher occurrence and variability of defensive actions, passes, and ball actions when there was a reduction in the field size and the number of players in U-8 [14], U-12 [9], and U-14 [13,25]. The use of the adapted rules format increased the number and variability of the players’ actions [13,25], which could involve a richer experience for the players.

The findings related to collective participation confirm previous observational studies in competition and training. A reduction in the field size and the number of players involved a higher number of offensive actions and their efficacy [9,10,13,26,27]. The reduction in the goal size increases goalkeeper participation due to a higher number of offensive actions [18]. Experimental studies in small-side games also found that the manipulation of the space and number of players are critical structural factors that affect the technical, tactical and physical actions of the players [11,28]. To our knowledge, this is the first experimental study that analyses the effect of these manipulations on the technical–tactical actions on competition. This type of study is critical to understand the impact of the rules format on the LTAD of youth football players. The competition is the reference of the process and involves critical experiences that affect player development. The competition rules are like the curriculum used in physical education to guide the process in order to achieve the desired competencies and skills. The rules format should be adapted to the maturity and skills of players, and it should provide a progressive, challenging, and achievable environment through the different stages of player development. However, in different age-groups, the adaptation of the formal rules reduces the participation and the variability and involves less efficacy in their actions; This may be due to the fact that the rules do not create technical–tactical situations appropriate for this age group [14]. These ideas have been proposed by previous observational studies done in different age groups [9,13,25].

The experimental rules tested promote the realization of passes, supporting the teammates, a higher number of actions close to the goals, a higher number of actions in defence and offence, and the use of the different zones of the field versus the format rules that promote the use of individual actions (e.g., dribbling) to a greater extent. This experimental format better fits players’ individual progression (U-12) and players’ and teams’ collective development. It could allow players to evolve from the individuality of previous stages, based on dribbling and side steps. With the experimental rules, there are fewer times that players dribble through the defensive lines, but more times that players pass through the defensive lines. The pass is a critical element for the tactical development of players and teams [29,30,31]. The experimental rules increase the use of the pass as a collective solution to the problems that come up in a game [32]. The use of different zones of the field introduces the concepts of generating space and centres in the game. These also allow one to introduce more complex tactical systems. The findings from this study and previous studies [9,10,18] show how a reduction in the field and goal size and the number of players in under-12 football provided a more appropriate experience for the players studied, due to the higher participation of the outfield players and goalkeepers, higher variability, and greater efficacy of their individual actions, and for the type of collective actions done.

The findings of the current study must be interpreted with caution. The study only analysed the short-term effects of the experimental rules. The medium- and long-term impact of training and competing with the experimental format is unknown. Future studies should analyse the impact of the rule modifications after a period of training. At this level, it must be considered that the use of the experimental format involves a reduction in the number of players per team, which could indirectly affect the players that play and the competition format. A possible solution to this problem could be to sub-divide the teams into two and play simultaneously on the reduced-sized field [33]. The study had a reduced sample, only male, and with specific sports skills (formative team). No teams from the elite U-12 teams of the region/zone were studied. This may influence the level of physical/biological development of the players, their skills, and their level of specialization. The findings show how the experimental rules allowed players to carry out more passes, have more variability in the way the actions are executed, and use different zones. This result can show the need to develop these skills in practice to continue their development (e.g., creating openings, wider use of the field, variability in the task, etc.). The study only analysed the technical and tactical actions of the players, and it did not analyse the physical actions, psychological variables, injuries, etc. Despite the limitations and delimitations, the findings show that the structural manipulation of the field and goal size and the number of players provided higher participation, variability, and tactical actions that could involve a better LTAD for the players studied. The current study is one of the first experimental steps in the study of rules formats and their impact on the LTAD of youth football players. Future studies should analyse the effect of the different rule formats in each age group, their synchronization, progression, and their relationship with the LTAD from a holistic perspective.

## 5. Conclusions

The experimental rules, namely the reduction in the number of players, field size, and goal size, promoted the realization of more actions and more variability in these actions. There was a significant increase in the passes that passed through defensive lines and a greater number of actions in the offensive zone. The experimental format better fits the players’ individual progression (U-12) and players’ and teams’ collective development, and it will allow the players to evolve from the individual development of previous stages. The rules of youth sport play a critical role in a player’s LTAD. Future studies are necessary to establish the proper rules for each age group to facilitate their appropriate development.

## Figures and Tables

**Table 1:** Description of the rules implemented in the tournaments (football-8 and football-5).

Rules	Official Rules (Football-8)	Modified Rules (Football-5)
Number of players	7 outfield players + 1 goalkeeper	4 outfield players + 1 goalkeeper
Number of players (team)	15	7
Field size (m)	58 × 38 m	38 × 20 m
Goal size (m)	6 × 2 m	3 × 2 m
Penalty area size (m)	24 × 9 m	12 × 6 m
Goal area size (m)	12 × 3 m	None used
Ratio of m^2^ per field player	314 m^2^	190 m^2^
m^2^ of the goal	12 m^2^	6 m^2^
Ball size (n)	4	4
Substitutions	Unlimited	Unlimited
Time (minutes)	2 × 20	2 × 20

**Table 2:** Technical-tactical actions through which the ball is received.

Player Action	Tournament 1 Football 8	Tournament 2 Football 5	Tournament 3 Football 8	Significance Post Hoc	Effect Size (*η^2^*)
Steal	5.84 ± 4.54	6.57 ± 6.09	6.14 ± 3.83	n.s.	0.054
Clearance	10.08 ± 5.40	14.14 ± 8.93	11.57 ± 5.76	T1 < T2 = T3	0.330
Rebound	1.46 ± 1.75	2.16 ± 2.19	1.57 ± 1.60	n.s.	0.119
Pass interception	10.54 ± 10.24	14.92 ± 11.68	12.19 ± 10.14	T1 < T2 = T3	0.295
Set-piece kick	3.84 ± 3.78	5.05 ± 4.15	3.86 ± 3.38	n.s.	0.109
Teammate Pass	20.76 ± 14.38	28.54 ± 20.20	24.78 ± 14.26	T1 < T2 = T3	0.339
Pass after throw-in	2.65 ± 2.77	4.84 ± 3.68	3.32 ± 2.79	T1 < T2 = T3	0.341
Pass after set piece	3.62 ± 3.41	4.30 ± 3.54	3.16 ± 2.91	n.s.	0.145
Throw-in	4.68 ± 8.08	9.62 ± 7.74	6.68 ± 11.23	T1 < T2 = T3	0.484

Legend: T1 = Tournament 1 (8 a-side); T2 = Tournament 2 (5 a-side); T3 = Tournament 3 (8 a-side); n.s. = no significant differences.

**Table 3:** Technical–tactical aspects in the obtaining of the ball.

Variable	Categories	Tournament 1 Football 8	Tournament 2 Football 5	Tournament 3 Football 8	Significance Post Hoc	Effect Size (*η^2^*^)^
Ball height	At ground level	32.73 ± 20.39	42.11 ± 30.96	36.97 ± 19.57	T1 < T2 = T3	0.263
	Middle ball	9.35 ± 5.86	11.92 ± 7.57	10.81 ± 4.80	T1 < T2 = T3	0.285
	High ball	11.00 ± 6.22	18.59 ± 11.16	13.38 ± 6.86	T1 < T2 > T3	0.443
Part obtaining the ball	Foot	49.22 ± 28.94	66.68 ± 41.52	56.78 ± 26.73	T1 < T2 = T3	0.384
	Hand	5.49 ± 8.48	9.86 ± 7.82	7.78 ± 11.17	T1 < T2 = T3	0.288
	Head	5.08 ± 4.65	9.65 ± 7.08	6.54 ± 5.47	T1 < T2 > T3	0.433
	Other	1.76 ± 1.99	3.27 ± 3.05	2.16 ± 1.80	T1 < T2 = T3	0.263
Distance from nearest opponent	Very close	16.41 ± 8.78	22.62 ± 16.71	19.92 ± 10.12	T1 < T2 = T3	0.287
	Close	13.68 ± 8.83	17.32 ± 12.33	15.92 ± 9.20	T1 < T2 = T3	0.250
	Near	10.70 ± 6.62	14.51 ± 8.40	11.03 ± 5.38	T1 < T2 > T3	0.281
	Far away	19.65 ± 13.16	37.08 ± 23.19	26.41 ± 19.87	T1 < T2 > T3	0.675
Teammates supporting	Yes	24.89 ± 15.57	38.19 ± 25.63	30.41 ± 19.81	T1 < T2 > T3	0.444

Legend: T1 = Tournament 1 (Football 8); T2 = Tournament 2 (Football 5); T3 = Tournament 3 (Football 8).

**Table 4:** Tactical aspects performed by the player with the ball in the development of the action.

Variable	Categories	Tournament 1 Football 8	Tournament 2 Football 5	Tournament 3 Football 8	Signific. Post Hoc	Effect Size (*η^2^*^)^
Collective tactical actions	No tactical actions	63.14 ± 33.97	88.83 ± 53.53	72.64 ± 35.71	T1 < T2 > T3	0.414
	Wall pass	1.58 ± 2.13	2.00 ± 3.08	0.061 ± 1.10	T1 = T2 > T3	0.314
	Others	0.25 ± 0.73	1.39 ± 1.53	0.11 ± 0.31	T1 < T2 > T3	0.409
Dribbles	No dribbling	50.89 ± 27.26	76.24 ± 43.72	60.32 ± 30.48	T1 < T2 > T3	0.483
	One	8.84 ± 7.49	10.70 ± 9.52	8.86 ± 6.43	n.s.	0.105
	≥2	4.35 ± 4.88	4.89 ± 5.35	4.08 ± 3.51	n.s.	0.054
Number of contacts in driving	No contacts	40.59 ± 23.59	58.22 ± 34.44	47.95 ± 26.77	T1 < T2 > T3	0.408
	2 contacts	13.92 ± 8.34	18.32 ± 12.95	16.22 ± 9.67	T1 < T2 = T3	0.272
	3–4 contacts	5.70 ± 5.75	7.22 ± 6.70	6.97 ± 5.96	n.s.	0.142
Type of dribble	There is no dribbling	40.19 ± 23.38	57.43 ± 33.50	47.81 ± 26.73	T1 < T2 > T3	0.406
	Timing	13.86 ± 8.41	19.68 ± 14.28	16.32 ± 9.69	T1 < T2 = T3	0.310
	Counterattack	9.65 ± 9.91	10.43 ±1 0.75	9.03 ± 7.36	n.s.	0.048
	Penetration	0.30 ± 0.66	4.30 ± 3.62	0.08 ± 0.27	T1 < T2 > T3	0.593
Pressure lines passed with ball	No pass line	33.59 ± 19.03	64.22 ± 32.78	48.05 ± 25.10	T1 < T2 > T3	0.713
	One line	19.54 ± 17.90	23.89 ± 22.75	22.49 ± 14.86	T1 <T 2 = T3	0.231
	More than one line	3.54 ± 3.83	3.73 ± 4.24	2.73 ± 2.21	n.s.	0.081

Legend: T1 = Tournament 1 (football-8); T2 = Tournament 2 (football-5); T3 = Tournament 3 (football-8); BCTAs: Basic collective tactical actions; n.s. = no significant differences.

**Table 5:** Technical-tactical actions by which players end ball possession.

Player Action	Tournament 1 Football 8	Tournament 2 Football 5	Tournament 3 Football 8	Significance Post Hoc	Effect Size (*η^2^*^)^
Pass to teammate	20.03 ± 14.27	26.65 ± 20.75	27.97 ± 15.79	T1 < T2 = T3	0.418
Lost pass	0.73 ± 1.40	0.89 ± 1.62	0.54 ± 0.93	n.s	0.075
Throw-in pass	1.62 ± 2.60	4.70 ± 3.82	1.32 ± 1.70	T1 < T2 > T3	0.569
Shot off target	3.38 ± 3.36	4.43 ± 3.49	3.24 ± 2.76	n.s.	0.144
Shot stopped by the goalkeeper	2.95 ± 3.13	6.73 ± 4.81	3.65 ± 3.22	T1 < T2 > T3	0.486
Ball lost	12.08 ± 7.92	16.24 ± 13.44	11.57 ± 7.48	T1 < T2 > T3	0.187
Clearance from a player	15.08 ± 12.03	22.95 ± 13.32	18.81 ± 14.57	T1 < T2 > T3	0.503
Out of bounds	2.68 ± 2.00	4.54 ± 3.71	2.43 ± 2.15	T1 < T2 > T3	0.307
Other action	2.86 ± 2.69	4.65 ± 3.90	3.49 ±2.99	T1 < T2 = T3	0.278

Legend: T1 = Tournament 1 (8 a side); T2 = Tournament 2 (5 a side); T3 = Tournament 3 (8 a side); n.s. = no significant differences.

**Table 6:** Technical–tactical aspects in the finalization of the possession of the ball.

Variable	Categories	Tournament 1 Football 8	Tournament 2 Football 5	Tournament 3 Football 8	Significance Post Hoc	Effect Size (*η^2^*)
Ball height	At ground level	30.89 ± 20.27	30.38 ± 23.59	35.62 ± 19.18	n.s.	0.112
	Middle ball	8.78 ± 5.01	23.32 ± 13.81	10.27 ± 5.26	T1 < T2 > T3	0.668
	High ball	24.38 ± 17.40	38.08 ± 23.80	27.35 ± 20.51	T1 < T2 > T3	0.399
Final body part in contact with the ball	Foot	50.68 ± 27.03	67.95 ± 41.53	58.97 ± 27.36	T1 < T2 = T3	0.351
	Hand	5.49 ± 8.11	9.84 ± 7.50	7.32 ± 11.01	T1 < T2 = T3	0.435
	Head	7.03 ± 5.21	12.59 ± 8.53	6.35 ± 4.68	T1 < T2 > T3	0.455
	Other	0.84 ± 1.16	1.46 ± 1.64	0.62 ± 0.75	T1 < T2 > T3	0.260
Distance from nearest opponent	Very close	25.81 ± 15.79	34.70 ± 24.44	27.46 ± 14.44	T1 < T2 > T3	0.271
	Close	14.51 ± 7.82	20.95 ± 12.40	18.24 ± 7.96	T1 < T2 = T3	0.434
	Near	11.11 ± 8.68	12.16 ± 9.24	9.62 ± 7.28	n.s.	0.133
	Far away	12.19 ± 11.40	24.03 ± 15.14	17.86 ± 16.75	T1 < T2 > T3	0.538
Teammates supporting	Yes	26.92 ± 17.67	38.59 ± 27.72	30.27 ± 16.26	T1 < T2 > T3	0.372
Pressure lines surpassed with ball	No pass line	38.57 ± 20.20	54.22 ± 30.69	47.19 ± 23.88	T1 < T2 = T3	0.385
	One line	22.00 ± 15.09	33.46 ± 24.79	23.27 ± 13.59	T1 < T2 > T3	0.428
	More than one line	3.57 ± 5.28	3.86 ± 3.83	2.78 ± 3.02	n.s.	0.128

Legend: T1 = Tournament 1 (8 a side); T2 = Tournament 2 (5 a side); T3 = Tournament 3 (8 a side); n.s. = no significant differences.
